# Comparison of Biomechanical and Microstructural Properties of Aortic Graft Materials in Aortic Repair Surgeries

**DOI:** 10.3390/jfb15090248

**Published:** 2024-08-28

**Authors:** Haoliang Sun, Zirui Cheng, Xiaoya Guo, Hongcheng Gu, Dalin Tang, Liang Wang

**Affiliations:** 1 Department of Cardiovascular Surgery, First Affiliated Hospital of Nanjing Medical University, Nanjing 210029, China; shlsky@126.com; 2 School of Biological Science and Medical Engineering, Southeast University, Nanjing 211189, China; ryanreal@foxmail.com (Z.C.); hcgu@seu.edu.cn (H.G.); dtang@wpi.edu (D.T.); 3 School of Science, Nanjing University of Posts and Telecommunications, Nanjing 210023, China; guoxiaoya1990@163.com; 4 Mathematical Sciences Department, Worcester Polytechnic Institute, Worcester, MA 01609, USA

**Keywords:** aortic graft material, aortic repair, mechanical testing, histological analysis, tissue microstructure

## Abstract

Mechanical mismatch between native aortas and aortic grafts can induce graft failure. This study aims to compare the mechanical and microstructural properties of different graft materials used in aortic repair surgeries with those of normal and dissected human ascending aortas. Five types of materials including normal aorta (n = 10), dissected aorta (n = 6), human pericardium (n = 8), bovine pericardium (n = 8) and Dacron graft (n = 5) were collected to perform uniaxial tensile testing to determine their material stiffness, and ultimate strength/stretch. The elastin and collagen contents in four tissue groups except for Dacron were quantified by histological examinations, while the material ultrastructure of five material groups was visualized by scanning electron microscope. Statistical results showed that three graft materials including Dacron, human pericardium and bovine pericardium had significantly higher ultimate strength and stiffness than both normal and dissected aortas. Human and bovine pericardia had significantly lower ultimate stretch than native aortas. Histological examinations revealed that normal and diseased aortic tissues had a significantly higher content of elastic fiber than two pericardial tissues, but less collagen fiber content. All four tissue groups exhibited lamellar fiber ultrastructure, with aortic tissues possessing thinner lamella. Dacron was composed of densely coalesced polyethylene terephthalate fibers in thick bundles. Aortic graft materials with denser fiber ultrastructure and/or higher content of collagen fiber than native aortic tissues, exhibited higher ultimate strength and stiffness. This information provides a basis to understand the mechanical failure of aortic grafts, and inspire the design of biomimetic aortic grafts.

## 1. Introduction

Aortic diseases including aortic dissection (AD), aortic aneurysm, and coarctation of the aorta among others can be life-threatening, and often require open-chest surgery to treat [1,2,3]. During aortic surgical interventions, aortic tubular grafts or patches made with synthetic materials or biological tissues are used to replace the diseased native aortic tissues [4]. Unfortunately, patients undergoing aortic replacement surgeries suffer late post-operative complications due to aortic graft failure, especially decades after surgery [5]. Long-term follow-up studies have shown that mechanical failure of aortic grafts such as graft dilation, aneurysm formation, restenosis and even graft rupture are not rare in clinical settings [6,7]. The mechanical mismatch between aortic grafts and native aortas is believed to be responsible for mechanical graft failure [8]. Therefore, characterizing the mechanical properties of these graft materials and comparing them with those of native aortic tissue are essential to understanding the pathological development of graft failures, and would also inspire the design of better aortic substitutes [9].

The choice of graft material used in aortic repair surgeries is highly associated with late complications requiring reoperations [4,10,11,12,13]. Ideally, an artificial aortic graft material should be biocompatible, durable, resistant to thrombosis and infection, easy to handle, readily available and low-cost [14], and should also match the mechanical properties of native aortic tissue [9]. Significant mechanical mismatch between aortic grafts and native aortas would result in excessive stresses at the anastomotic sites, leading to intimal hyperplasia, tissue fatigue, or anastomotic aneurysm and even rupture [9,11]. Furthermore, inelastic graft materials could lead to unwanted hemodynamic alterations in the cardiovascular system, like inducing additional workload for the left ventricle, and increasing the systolic flow to the distal aorta [12,13]. Even though the mechanical mismatch has profound impacts on the prognosis of aortic replacement surgery, few studies exist to determine the mechanical properties of graft materials and compare them to the native aortic tissues in normal or diseased [14,15]. Using biaxial tensile testing, Tremblay et al. compared the mechanical properties of healthy aortic tissues, dilated aortic tissues and three other aortic graft materials. Significant differences in material stiffness and anisotropy were found among all material types [14]. To characterize the failure properties of the aortic grafts, Recco et al. compared the ultimate strength of three aortic graft materials. They found that the failure strength of pulmonary homograft was significantly smaller than that of autologous pericardium and bovine pericardium [15]. However, a direct comparison with native aortic tissues was lacking in the latter study.

When studying the mechanical properties, the microstructural properties of the graft materials should also be considered to thoroughly understand their material behaviors [16]. Much progress has been made in revealing the relationship between mechanical and structural properties in cardiovascular tissues. An experimental animal study demonstrated that the collagen fiber in the murine aorta modulated both material stiffness and strength of the vessel wall, and contributed to the increased stiffness in aneurysmal samples [17]. This relationship was further investigated by Sherifove et al. as they reported that the failure stresses in human aortic samples were inversely associated with the collagen fiber direction relative to the loading axis [18]. This suggested that the magnitude of the failure stress was in part attributed to the collagen architecture. Pukaluk et al. investigated the microstructural changes of the human atherosclerotic abdominal aortic media under biaxial loading using multi-photon microscopy. Their observation of microstructural alterations could provide an explanation of the exhibited mechanical behavior of the aortic media [19]. A similar approach was applied to illustrate the structural–mechanical relationship of the human aortic adventitia [20]. All this evidence has shown that the material properties are closely associated with the tissue microstructure [16]. However, the microstructural and mechanical properties of aortic grafts were not simultaneously investigated in the current literature. Therefore, this paper aims to compare the mechanical and microstructural properties of five materials including native aortic tissues and different aortic grafts, to better understand the mechanical failure of aortic grafts, and to provide a basis for further optimizing the design of biomimetic aortic grafts.

## 2. Materials and Methods

### 2.1. Sample Collection and Preparation

Five materials including normal ascending aortic tissue (NA group), diseased ascending aortic tissue from patients with type A aortic dissection (AD group), human pericardial tissue (HP group), bovine pericardial tissue (BP group) and synthetic Dacron graft (Dacron group) were collected for comparison purposes. All human tissue specimens were harvested at Jiangsu Province Hospital with informed consent obtained. After resection, the tissue specimens were preserved in a cryopreservation solution (85% RPMI 1640 culture medium, 5% albumin solution (20%), and 10% dimethyl sulfoxide) in a −80 °C freezer [21]. Prior to the mechanical and microstructural characterizations, the tissue specimens were thawed in phosphate-buffered saline solution at room temperature until they were completely defrosted. The study was performed following the protocol approved by the Medical Ethics Committee of Jiangsu Province Hospital (approval number: 2022-SR-730). Details on the sample preparation in each material group are as follows:

Normal ascending aortic specimens were acquired from 10 organ donors (8 males/2 females, age: 43.9 ± 12.9) without any aortic diseases. Two dog-bone shape tissue samples were prepared from each specimen for uniaxial tensile testing [22], with one sample in the circumferential direction and one in the longitudinal direction.

Diseased ascending aortic specimens were collected from six patients with type A aortic dissection (4 males/2 females, age: 52.5 ± 11.9) during the aortic replacement surgery following Sun’s procedure [23]. In total of 12 samples were cut from the non-dissected regions with seven in circumferential and five in longitudinal direction.

Human pericardial specimens were also harvested from eight organ donors (5 males/3 females; age: 58.7 ± 6.1) to obtain 16 dog-bone shape samples. The 16 samples were split into two subgroups by selecting one sample in every two samples to be chemically treated with 0.625% glutaraldehyde for 10 min (fixed, n = 8), and the other one in fresh state (fresh, n = 8).

Commercially available bovine pericardium specimens (Beijing Balance Medical Technology Co., Ltd., Beijing, China) were obtained to prepare 19 dog-bone shape strips from 8 specimens. Sample direction was not recorded since the bovine pericardium product was in a rectangular shape with no direction information indicated.

Five specimens of woven double velour Dacron graft (InterVascular SAS, La Ciotat, France) were also prepared to obtain 10 dog-bone shape strips, with one sample in each direction from each sample.

All dog-bone shape strips are about 30 mm × 5 mm, with the narrowest part of width 2 mm. The thickness of all samples was measured at four different locations, and the average value was taken as sample thickness.

### 2.2. Uniaxial Tensile Testing

**Figure 1:**
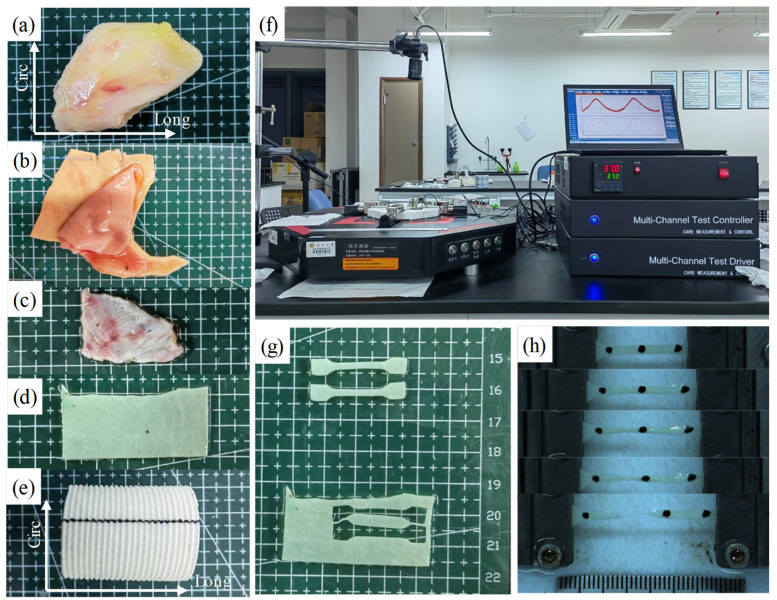
Sample preparation for uniaxial tensile testing. (**a**–**e**) Specimens of normal aorta (**a**), dissected aorta (**b**), human pericardium (**c**), bovine pericardium (**d**) and Dacron graft (**e**); (**f**) Mechanical testing system for uniaxial tensile testing; (**g**) tissue preparation in dog-bone shape; (**h**) Recorded images showing the testing process of a sample to material failure. Sample directions (Circ for circumferential direction; Long for longitudinal direction) were indicated in (**a**,**b**,**e**).

All dog-bone shape samples were then mounted into a mechanical testing system (IPBF-300, CARE Measurement and Control) using clamps to perform the uniaxial tensile testing (see Figure 1f) [21]. The system is equipped with a load cell and displacement transducer to measure the force and displacement data. Uniaxial tensile testing was carried out on all dog-bone shape samples following a standard procedure established in previous literature [15,24,25]. To reduce tissue hysteresis, the samples were mechanically preconditioned by executing five loading–unloading cycles at a constant speed of 0.1 mm/s to a maximum displacement of 2 mm. Then the real testing was performed with the same speed of 0.1 mm/s and a pre-loading of 0.01 N until material failure occurred [24]. To better mimic the in vivo conditions, the samples were submerged in the 37 °C phosphate-buffered saline bath during the testing process [25].

### 2.3. Constitutive Modeling

The force and displacement data were used to derive the stress and stretch ratio data [23,26]. To characterize the mechanical failure properties of these materials, the stress and stretch values corresponding to the material failure point were recorded and denoted as ultimate stress and stretch, respectively. For constitutive modeling, tissue samples were assumed to be an incompressible homogeneous hyperelastic material. Modified isotropic Mooney–Rivlin model with the following strain energy density function were employed to fit the stress–stretch data before material failed [26,27]:

W = c_1_(I_1_ − 3) + D_1_{exp(D_2_(I_1_ − 3)) − 1}, (1)

where I_1_ = ∑C_ii_ is the first invariants of right Cauchy–Green deformation tensor C = [C_ij_] = X^T^X, X = [X_ij_] = [∂x_i_/∂a_j_], (x_i_) is the current position, (a_i_) is the original position. c_1_, D_1_ and D_2_ are material parameters. A trust-region-reflective algorithm was used to determine the material parameters, with the coefficient of determination (R^2^ value) to evaluate the goodness of fit [28]. To compare the material stiffness among different sample groups, effective Young’s modulus [21] was defined as the slope of the proportional function to fit the material curve on the stretch interval [1.0, 1.3] to measure the material stiffness, given that human aorta typically works in this stretch range under the physiological conditions [29].

### 2.4. Quantitative Histological Analysis

**Figure 2:**
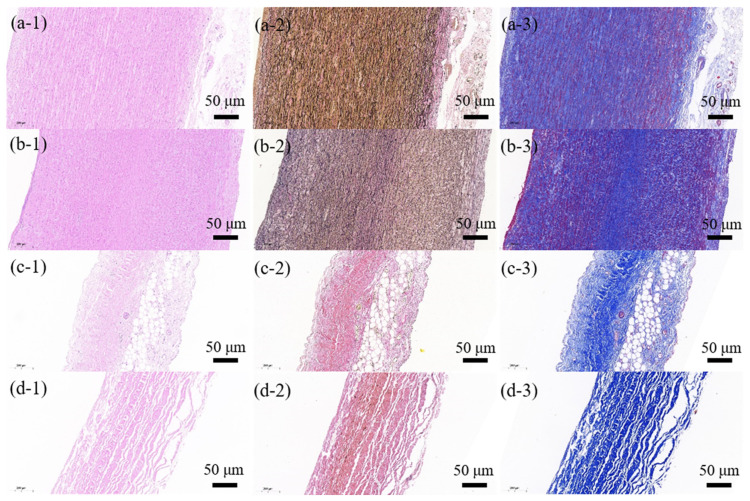
Representative HE, EVG and Masson images of four sample types including normal aortic tissue (**a1**–**a3**), diseased aortic tissue (**b1**–**b3**), human pericardial (**c1**–**c3**) and bovine pericardial tissues (**d1**–**d3**). All scale bars are 50 µm.

Along with the mechanical testing, an additional sample was cut from each specimen from the location adjacent to the testing samples to perform the histological analysis. The sample was fixed in formalin for 24 h, dehydrated through a process of varied alcohol concentrations, and then embedded in paraffin and serially sectioned into a 5-μm thickness section [30,31]. The tissue histology was visualized by staining the consecutive sections using Elastin van Gieson (EVG) for black-stained elastic fiber, Masson’s trichrome for blue-stained collagen fiber, and hematoxylin–eosin (HE) for gross tissue morphology, respectively [26,30]. All samples were stained in one batch for each histological staining to minimize any batch effects, and then histological slides were scanned with a digital slide scanner (Pannoramic MIDI, 3DHISTECH, Budapest, Hungary). The contents of elastic and collagen fibers in tissue samples defined as the areal percent occupied by each stained fiber over the entire tissue sample [31,32], were extracted and quantified using the threshold value algorithm from EVG images and Masson images, respectively [26,32]. It is worth noting that Dacron is made from polyethylene terephthalate (PET) fiber, which does not contain the biological elastin and collagen fibers, so its histological analysis was not performed [33]. Figure 2 shows the HE, EVG and Masson images of four tissue types. More details on the image processing of the EVG and Masson images are provided in Supplementary Materials Figure S1.

### 2.5. Structural Characterization

To compare the ultrastructure of five material groups, a field emission scanning electron microscope (SEM) (Ultra Plus, Zeiss, Oberkochen, Germany) was utilized to capture the high-resolution images of their surface morphologies. Only one sample was prepared for each group to examine the ultrastructure of the cross-section of the material. Tissue sample was frozen at −80 °C for 24 h, followed by removing the ice in the tissue by sublimation in a vacuum evaporator [34]. After coating the sample in gold to be electrically conductive, the microscope was operated using an accelerating voltage of 1.2 kV and a working distance of about 5 mm to visualize its tissue ultrastructure [35].

### 2.6. Statistical Analysis

Due to the small sample size in each material group, mechanical or microstructural data do not satisfy the normality assumption after checking with the Shapiro–Wilk test. Thus, continuous variables were reported as median [interquartile range]. The nonparametric Kruskal–Wallis H test was employed to determine whether differences exist among five material groups with the Tukey–Kramer test adopted for the post-hoc test for any pair of two groups. Moreover, Mann–Whitey U test was used to test whether there is a significant difference between two sample directions in one material group, or between fresh and fixed subgroups in the human pericardium group. Statistical analysis was performed with MATLAB (MathWorks Inc., Natick, MA, USA) with a statistical significance level of 0.05.

## 3. Results

### 3.1. Comparison of Mechanical Failure Properties among Five Material Groups

**Figure 3:**
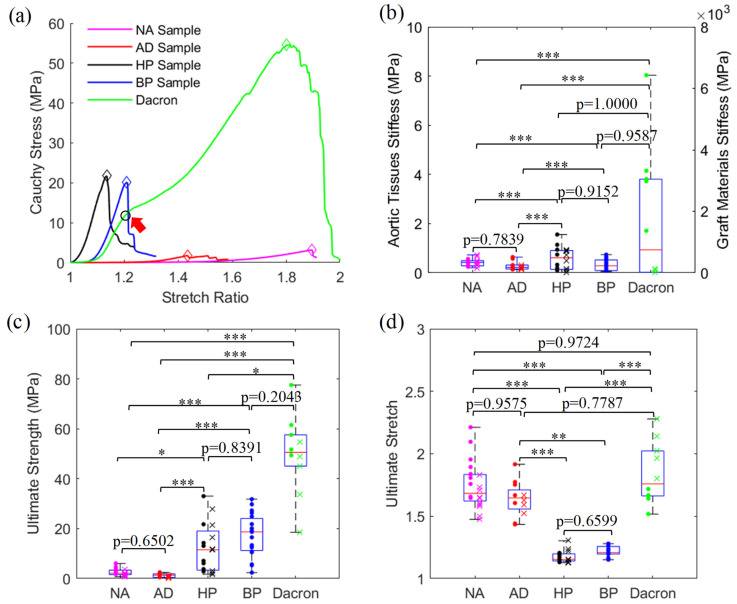
Comparison of mechanical properties among five material groups. Representative material curves (**a**), material stiffness (**b**), ultimate stress (**c**), and ultimate stretch (**d**). The red arrow in (**a**) marks the end of the first segment of material curve of Dacron. The data from the sample in the circumferential direction (or fresh human pericardial sample) were presented as dot markers while data from the longitudinal direction (or fixed human pericardial sample) as a cross marker (* means *p* < 0.05, ** means *p* < 0.01, *** means *p* < 0.001).

The representative stress–stretch curves of samples in five material groups are provided in Figure 3a. Most samples exhibited nonlinear J-shape material curves, indicating the samples became stiffer as the stretch level increased. However, Dacron material showed a very special mechanical behavior in that its material curve is not a smooth J-curve before reaching the material failure point, and can be seen as a combination of multiple segments of smooth J-curves.

The ultimate strength and stretch data of all samples are provided in Figure 3c,d. There were significant differences in ultimate strength and stretch among the five material groups (both *p* < 0.0001). The failure strength was highest in Dacron material (50.6 [45.1, 57.6] MPa), and decreases in the order of BP (18.7 [11.2, 24.1] MPa), HP (11.5 [3.39, 19.0] MPa), NA (2.12 [1.56, 3.37] MPa) and AD samples (1.37 [0.475, 1.85] MPa), all statistically significant (*p* < 0.05) except for the differences between BP and HP or Dacron samples (Figure 3c). Intra-group comparison was also performed to demonstrate that the failure strength in the circumferential direction was higher than that in the longitudinal direction for the NA group (*p* = 0.0017), AD group (*p* = 0.0303) and Dacron group (*p* = 0.0317).

For ultimate stretch, Dacron material also had a very high ultimate stretch (1.76 [1.66, 2.02]), but it is close to the ultimate stretch of those from NA (1.68 [1.62, 1.83]) and AD tissues (1.65 [1.55, 1.71]) with no statistical difference found. BP (1.21 [1.20, 1.25]) and HP (1.15 [1.14, 1.20]) had similar failure stretch levels, but their values were significantly lower than those from the other three groups. The intra-group comparison showed that the ultimate stretch in the circumferential direction was significantly higher than that in the longitudinal direction for the NA group (*p* = 0.0058), but significantly lower for Dacron material (*p* = 0.0079). No significance was found in the failure stretch between the two directions of the AD group (*p* = 0.6389). As to HP tissues, both ultimate stress and stretch were not significantly impacted by the fixation process (*p* = 0.7209 and *p* = 0.7984).

### 3.2. Comparison of Material Stiffness among Five Material Groups

The material parameters of the Mooney–Rivlin model were determined by fitting the experimental stress–stretch data for all tissue samples. The average R^2^ value over all samples is 0.9749, demonstrating that this model can accurately capture the mechanical behaviors of all five material types. As can be seen in Figure 3b, Dacron had the highest material stiffness (748 [18.2, 3048] MPa), which was significantly higher than both NA (0.423 [0.283, 0.494] MPa) and AD groups (0.222 [0.153, 0.316] MPa), but not significantly stiffer than HP (490 [111, 723] MPa) and BP groups (30 [69.8, 419] MPa), due to the huge difference between two directions in Dacron group (circumferential: 3048 [2576, 4096] MPa; longitudinal: 18.2 [3.99, 96.0] MPa, *p* = 0.0079). For the rest four tissue types, HP was numerically stiffer than BP, but the difference was not significant (Figure 3b). Both pericardial tissues were significantly stiffer than aortic tissues, either in normal or diseased states. For direction-specific material stiffness, no significant difference was found in the NA (*p* = 0.1041) and AD groups (*p* = 0.5303). Lastly, the fixation process also had no significant impact on the stiffness of human pericardial tissues (*p* = 0.5737).

### 3.3. Comparison of Histological Properties among Four Tissue Groups

**Figure 4:**
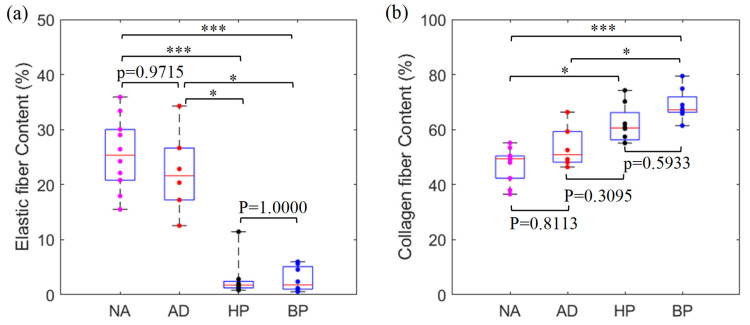
Comparison of elastic (**a**) and collagen (**b**) fiber contents among four tissue groups. The data from each specimen were presented as one dot (* means *p* < 0.05, *** means *p* < 0.001).

Histological staining was performed on each specimen of four tissue groups other than Dacron material. The gross examination showed that normal and diseased aortic tissues exhibited lamellar elastic fiber structure, with the lamellar structure in AD group more disorganized and disrupted, compared to the NA group (see Figure 2). Compared to aortic tissues, HP and BP tissues also presented lamellar fiber structure, mostly consisting of collagen fiber and much less elastic fiber. Statistical analysis confirmed that NA or AD tissues contained similar amounts of both fiber contents. They had a significantly higher amount of elastic fiber than HP/BP tissues, but less collagen fiber than only BP tissues (see Figure 4).

### 3.4. Tissue Ultrastructure from SEM

**Figure 5:**
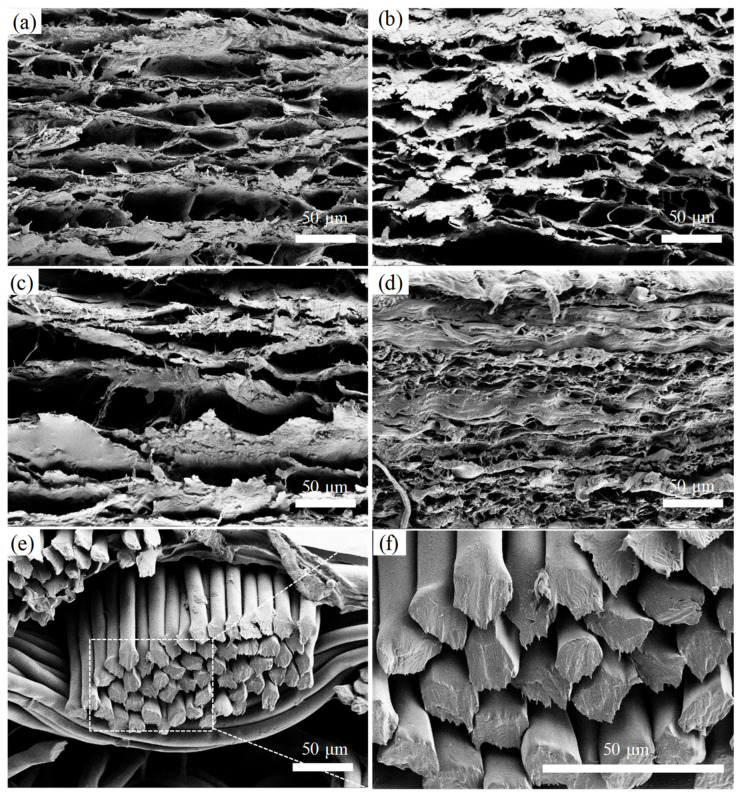
Tissue ultrastructure of five materials using SEM. (**a**) Normal aortic tissue; (**b**) Diseases aortic tissue; (**c**) Human pericardial tissue; (**d**) Bovine Pericardial tissue; (**e**,**f**) Dacron material. Magnification ×1000 for (**a**–**e**), ×3000 for (**f**). All scale bars are 50 µm.

The ultrastructure of the cross-sectional of five materials from SEM is provided in Figure 5. NA or AD tissues had a thin lamellar structure, with the cross-linking fibers forming irregular clefts [36]. They presented more clefts between the fiber structures, compared to the pericardial tissues. Both HP and BP also showed lamellar ultrastructure, but the lamella was thicker than that in aortic tissues. BP exhibited a more densely packed fiber structure than HP, with almost no visible distance between lamellas. For Dacron material, it consists of coalesced PET fibers in thick bundles. This material presented most dense fiber structure than the other four types of tissues.

## 4. Discussion

### 4.1. Clinical Implication from Mechanical Comparison among Five Materials

Aortic graft or patch material selection for aortic reconstruction historically has been based on the surgeon’s preference without quantitative mechanical and microstructural information of graft materials to support the graft selection [15,37]. Even though not common, mechanical failure of aortic graft like aneurysm formation or rupture at the repair sites [38] imposes life-threatening conditions on the patients after aortic reconstruction surgery [7]. The replacement of the native aorta with graft materials would result in alterations in the biomechanical stress/stretch and hemodynamics in the aorta which could have consequential effects on the graft integrity [15]. Therefore, it is essential to obtain the mechanical behaviors of these materials to advance our understanding of mechanical graft failures [10].

The mechanical properties of graft materials are closely related to their performance in vivo [12]. From a mechanical point of view, graft rupture occurs when the mechanical stress induced by the external loading exceeds the graft material strength [10]. Knowledge of material strength may help prevent the occurrence of graft or patch rupture, especially in the immediate postoperative period when the material is most vulnerable to rupture [15]. Our results showed that all three graft materials had significantly higher material strength than normal aortas. More specifically, Dacron material had the highest median value of ultimate strength, about 24 times higher than that of normal aortic tissues. BP and HP tissues had about four to eight times higher median values of ultimate strength than normal aortic tissues. Higher material strength in three graft materials showed that they are more resistant to rupture than native aortas. This may explain the clinical observation that graft rupture is less common than other mechanical graft failures, like aneurysmal dilatation and stenosis [10].

Ultimate stretch is also believed to be associated with material failure, as examined in various cardiovascular tissues [39,40]. A uniaxial tensile study revealed that tissue rupture in carotid atherosclerotic plaque initially occurred within the region undergoing a higher stretch ratio [40]. Our results showed that, compared to aortic tissues, HP and BP samples can withstand relatively large stress conditions (ultimate strength around 10–20 MPa), but they cannot bear large stretch, and normally fail at a stretch level below 1.3. This means that the two types of materials should be used cautiously in a setting for patients with large stretch in vivo. On the other hand, Dacron material had a higher median value of ultimate stretch, close to that of normal aortas. Therefore, according to the ultimate strength and stretch data among the three grafts, Dacron tends to be a safer choice in regard to the occurrence of aortic graft rupture.

It can be observed in Figure 3a that the synthetic Dacron material had a stress–stretch curve with multiple stages. The shape of the material curve obtained in this study is typical of Dacron material in general and confirms the results from previous studies [29,41]. Actually, a microstructural model was developed to explain the shape of the material curve [41,42,43]. In the model, PET fiber is made up of microfibril which further consists of amorphous regions and crystalline blocks. The dynamic alterations in these microstructural components, such as alignment of the amorphous phases, straightening of amorphous regions and the slippage in crystalline blocks are accounting for multiple stages of the material curve [42,43]. These studies also suggested that this material started yielding at a very low stress value, compared to its ultimate strength [33,42,43]. Long-term cyclic stretch of the material under in vivo conditions would lead to graft fatigue, which may explain why the Dacron graft also had a high incidence of graft aneurysm, even though its ultimate stress and stretch are quite high [7].

Besides the material failure properties, a huge difference in material stiffness between these materials was also observed in this study. Clinical follow-up studies have accumulated evidence that mechanical mismatch, more specifically, material stiffness mismatch between the aortic graft and native aorta resulted in long-term unwanted clinical outcomes in graft implantations [8]. The mechanical mismatch could impart excessive stresses at the suture lines, resulting in an anastomotic aneurysm [8,10,11], and also induce deleterious hemodynamic effects in cardiovascular hemodynamic, such as systolic hypertension [37,38]. These unwanted hemodynamic changes would promote platelet accumulation at distal anastomosis. Together with the proliferation and migration of smooth muscle cells due to the excessive stresses at the suture lines, intimal hyperplasia formation would occur, and eventually lead to the stenosis in the graft [10]. A direct comparison study reported that stenosis was presented in 70% of patients after aortic arch reconstruction with bovine pericardium while presented in 23% of patients with pulmonary artery or aortic homograft [44]. Furthermore, the mechanical mismatch is also responsible for long-term graft size change. Takami et al. reported that the diameter of Dacron grafts used in the ascending aorta increased by around 26% immediately after implantation, and dilated gradually at 3.2% per year in diameter after implantation [45].

Graft material selection is multifaceted and includes consideration of several crucial factors. Hopefully, the mechanical comparison analysis would make surgeons more aware of the significant differences in mechanical properties and their potential effect on the treatment outcome [14].

### 4.2. Relationship between Mechanical and Microstructural/Ultrastructural Properties

Gross examination of the SEM images showed that all four tissue groups exhibited lamellar structure, with more clefts between fiber structures in aortic tissues. Combining SEM and histological stain images, the lamellar structure in aortic tissues should be elastic fiber together with collagen fiber. While in pericardial tissues, lamellar structure is mainly composed of collagen fibers. The elastic and collagen fibers are two important microstructural compositions regarding the mechanical properties of cardiovascular tissues. At the micro level, elastic fiber is relatively compliant and stretchable, but cannot bear large tension (stiffness~1 MPa, ultimate stretch~2.0, ultimate strength~1 MPa) while collagen fiber is tough and much stiffer, but unstretchable (stiffness~1.0 GPa, ultimate stretch~1.13, ultimate strength~100 MPa) [9]. Further macro-level investigations have confirmed that the elastic fiber of the vascular tissues contributes to its extensibility and the collagen fiber provides the material strength [46,47,48]. Therefore, it is reasonable to infer that the pericardial tissues mainly consist of collagen fibers and would have high ultimate strength but low ultimate stretch based on their microstructural constitutions. They are also much stiffer than aortic tissues. Moreover, these two fibers are also responsible for the J-shape material curves of these aortic and pericardial tissues. An experimental study conducted by Roach and Burton has illustrated that at low tension or pressure conditions, the elastic fibers stretch to resist the applied loading, while the collagen fibers are in wavy state, and cannot bear the mechanical loading [46]. At higher tension or pressure conditions, more collagen fibers start to straighten, and become the predominant fiber structure to resist the applied loading. That is why the material stiffness of these cardiovascular tissues increases as the applied loading elevates.

Dacron is a type of woven synthetic material composed of PET fibers. Compared to the biological elastic and collagen fibers, PET fibers had a material strength of about 1.0 GPa [42], which is comparable to that of collagen fibers, and significantly stiffer than elastic fibers. However, Dacron material offers a significantly higher strength than pericardial tissues, which are mainly composed of collagen fibers. The difference may be in part attributed to the fiber microstructure. SEM images showed that Dacron material is densely filled with PET fibers in thick bundles. As the previous study showed that the porosity is inversely related to its mechanical strength in some biomaterials such as bone scaffold material [49], it is natural to think that the high fiber volume fraction in Dacron would contribute to its high material strength. It is worth noting that the fabrication process and woven techniques may also impact the material properties, as can be seen in the huge difference of Dacron samples between circumferential and longitudinal directions [33].

### 4.3. Mechanical–Microstructural Property Relationship for Aortic Graft Design

An ideal aortic conduit should not only offer adequate structural, biological and mechanical properties, but also be cost-efficient, and easy to handle in clinical practices. Unfortunately, none of the available aortic grafts satisfy all the requirements [50]. With an aim to overcome the current disadvantages of artificial grafts, great efforts have been exerted on the bioengineering front to pursue novel biomimetic aortic substitutes that can maintain long-term vascular patency in vivo [13]. An aortic graft made of equine pericardial tissue was developed for arch reconstruction or aortic root enlargement in a clinical setting, but patch rupture or stenosis could happen at follow-up [34,50]. Further, bioresorbable patches were also designed, and the patch was currently applied to treat the aortic dissections in the animal model, and is still in its fancy for clinical applications [51].

Recent studies are more centered on generating biomimetic grafts capable of reproducing the mechanical properties of native aortas. Our comparison analysis may shed some light on producing a vascular graft based on the mechanical–structural property relationship. As can be seen in this study, the ultimate stretch data of four tissue groups had the trend of NA > AD > BP > HP (Figure 3d), which was the same trend in elastic fiber content in the four tissue groups. This suggested that the extensibility of aortic or graft tissues can partially be attributed to the elastic fiber content. Furthermore, the pericardial tissues with higher collagen fiber content offered higher material stiffness and strength than aortic tissues. The evidence implies that varying the elastic and collagen fiber contents and their cross-linking architectures may hold the potential to modulate the mechanical properties of vascular scaffolds for aortic graft tissue engineering.

### 4.4. Study Limitations

(1) Histology. The quantitative measurements of elastic and collagen fiber contents depend on the sampling locations for histological staining. Care was taken to select the site adjacent to the sample sites for tensile testing to perform histological staining; (2) Uniaxial tensile testing was performed to determine the mechanical failure properties. Biaxial testing should be performed to obtain more through anisotropic material properties of the grafts [14]; (3) For some tissue types, like human and bovine pericardium, the sample direction, like circumferential or longitudinal directions cannot be identified, and were not considered in this study; (4) Other synthetic materials like Teflon or Gore-Tex materials should be included to perform the mechanical and microstructural characterizations for comparison purposes when available. (5) The sample size is relatively small in our study. Large sample size studies are needed for further validation.

## 5. Conclusions

There is a significant difference in mechanical and microstructural properties between native aortic tissues and graft materials. Compared to native aortic tissues, both human and bovine pericardial tissues possessed less elastic fibers and more collagen fibers, exhibiting higher ultimate strength and stiffness, but less extensibility. Dacron material made up of dense PET fiber ultrastructure, held the highest ultimate strength and stiffness among all five materials. Comparison analysis of these materials would advance our understanding of the occurrence of mechanical graft failure in clinical studies, and also provide important information on graft structural–mechanical relationship for graft design optimization.

## Data Availability

The raw data supporting the conclusions of this article will be made available by the authors on request.

## Supplementary Materials

The following supporting information can be downloaded at: https://www.mdpi.com/article/10.3390/jfb15090248/s1, Figure S1: Illustration of the image processing to calculate the elastic and collagen fiber contents in normal aortic specimen (**a**), diseased aortic specimen (**b**), human pericardial specimen, and bovine pericardial specimen (**d**). ((**a**–**d**)-1) Original EVG image; ((**a**–**d**)-2) Segmented elastic fiber. Background area in white; Tissue area in gray; Elastic fiber in black; ((**a**–**d**)-3) Original Masson image; ((**a**–**d**)-4) Segmented collagen fiber. Background area in white; Tissue area in gray; Collagen fiber in blue. All scale bars are 50 µm.
