# Supplementary material for: Comparison of Biomechanical and Microstructural Properties of Aortic Graft Materials in Aortic Repair Surgeries
Source: J Funct Biomater. 2024 Aug 28;15(9):248. doi: 10.3390/jfb15090248 (PMC11433388; doi:10.3390/jfb15090248)
Supplement: Supplementary file 1 [file jfb-15-00248-s001.zip › jfb-3099651-supplementary.pdf]

Journal of Functional Biomaterials

MDPI

Article

Comparison of Biomechanical and Microstructural Properties of Aortic Graft Materials in Aortic Repair Surgeries

Haoliang Sun [1], Zirui Cheng [2], Xiaoya Guo [3], Hongcheng Gu [2], Dalin Tang [2,4] and Liang Wang [2,*]

1 Department of Cardiovascular Surgery, First Affiliated Hospital of Nanjing Medical University, Nanjing 210029, China; shlsky@126.com
2 School of Biological Science and Medical Engineering, Southeast University, Nanjing 211189, China; ryanreal@foxmail.com (Z.C.); hcgu@seu.edu.cn (H.G.); dtang@wpi.edu (D.T.)
3 School of Science, Nanjing University of Posts and Telecommunications, Nanjing 210023, China; guoxiaoya1990@163.com
4 Mathematical Sciences Department, Worcester Polytechnic Institute, Worcester, MA 01609, USA
* Correspondence: liangwang@seu.edu.cn; Tel.: +86-158-5056-0404

Histological Analysis for Elastic and Collagen Fiber Densities

Elastin van Gieson (EVG) staining and Masson's trichrome staining were performed to visualize the contents of the elastic and collagen fibers for each tissue specimen from four tissue groups except Dacron material. The elastic fiber was stained in black on EVG image while collagen fiber was stained in blue on Masson image. For each image, the areas for the two fibers were determined by the threshold value to segment for the areas for elastic and collagen fibers from EVG and Masson images, respectively (see Figure S1a-d). After deleting the background region, the areal percent of each fiber was calculated as the ratio of fiber area over the total tissue area. The imaging process was implemented with MATLAB (MathWorks).

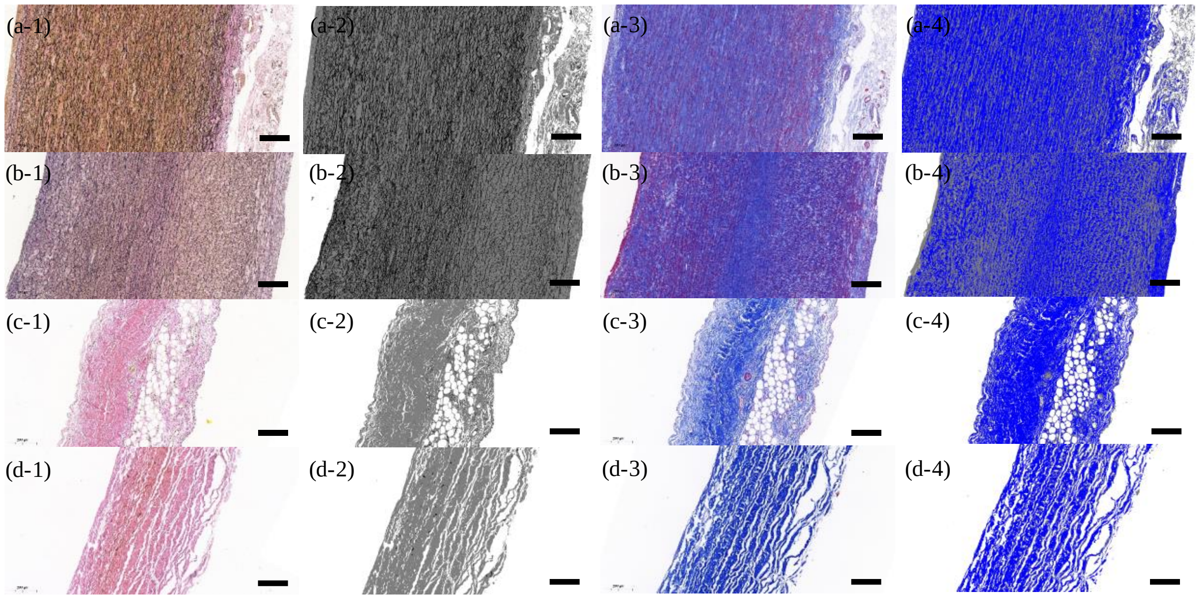

Figure S1. Illustration of the image processing to calculate the elastic and collagen fiber contents in normal aortic specimen (a), diseased aortic specimen (b), human pericardial specimen, and bovine pericardial specimen (d). ((a-d)-1) Original EVG image; ((a-d)-2) Segmented elastic fiber. Background area in white; Tissue area in gray; Elastic fiber in black; ((a-d)-3) Original Masson image; ((a-d)-4) Segmented collagen fiber. Background area in white; Tissue area in gray; Collagen fiber in blue. All scale bars are 50 μm.
